# Effect of He Plasma Jet Versus Surface Plasma on the Metabolites of Acute Myeloid Leukemia Cells

**DOI:** 10.3389/fonc.2021.552480

**Published:** 2021-03-17

**Authors:** Dehui Xu, Ning Ning, Yujing Xu, Wenjie Xia, Dingxin Liu, Hailan Chen, Michael G. Kong

**Affiliations:** ^1^ State Key Laboratory of Electrical Insulation and Power Equipment, Centre for Plasma Biomedicine, Xi’an Jiaotong University, Xi’an, China; ^2^ The School of Life Science and Technology, Xi’an Jiaotong University, Xi’an, China; ^3^ Frank Reidy Center for Bioelectrics, Old Dominion University, Norfolk, VA, United States; ^4^ Department of Electrical and Computer Engineering, Old Dominion University, Norfolk, VA, United States

**Keywords:** cold atmospheric plasma, acute myeloid leukemia, He plasma jet, surface plasma, glutamine, glutaminase, alanine, aspartate and glutamate metabolism

## Abstract

Cold atmospheric plasma, including plasma jet and surface plasma, can promote the apoptosis of cancer cells without causing significant damage to surrounding normal cells, which was hopeful to be applied to the clinical cancer therapy. However, experimental plasma devices used directly to clinical experiments has challenges in technology and methods, especially the difference in killing tumor cells efficiency of these two common plasma sources. Therefore, it is great necessity to explore the differences in treating tumors between different plasma sources. This paper achieved good killing efficiency by using two kinds of cold atmospheric plasma generating devices, namely plasma jet and surface plasma treatment along acute myeloid leukemia (AML). The results showed that the He plasma jet kills leukemia cells more efficiently than surface plasma with the same voltage and frequency and the same time. By GC-TOFMS and metabolomics analysis, this paper compared the differential metabolites of leukemia cells treated by two plasma devices and the key metabolic pathways closely related to differential metabolites. Simultaneously, we found alanine, aspartate and glutamate metabolism was most correlated with a key differential metabolite, glutamine. It was found that the glutaminase activity of He plasma jet group was lower than that of surface plasma group, which might be a reason for He plasma jet group to kill tumor cells better. It was also worth noting that relative quantity of glucose metabolites of plasma jet treatment group was lower than that of surface plasma treatment group. This study provides the basis for clinical trials for future.

## Introduction

Cold atmospheric plasma (CAP) is a groundbreaking technique that overcomes the limits of thermal plasma and reduces the gas temperature to room temperature so that the cold plasma can be used directly to handle biological tissue (1–3). Thus, the application of cold plasma in the medical and biological fields has been developed rapidly in recent years. The most commonly used applications include sterilization, wound healing, dermopathic treatment, and cancer treatment (4–12). It has been reported that CAP cam efficiently kill various types of tumor cells, including lung cancer, leukemia, intestinal cancer, melanoma, cervical cancer, glioma, and pancreatic cancer (13–20). There is no doubt that plasma medicine has obtained great research results and many new discoveries in the field of cancer treatment, and it is hopeful that the research results of laboratory plasma *in vitro* and *in vivo* treatments will eventually be used in clinical therapy of cancer (21–23). However, the direct application of plasma has double challenges in technology and method, especially the difference in the killing effect of different types of plasma sources on tumor cells. The same power supply parameters may achieve different therapeutic effects. Therefore, it is of great significance to explore the differences in the effects of different plasma sources on tumor treatment. Cold atmospheric plasma could generate aqueous reactive species including OH, H_2_O_2_, O_3_, nitrite $\left(\text{HN}{\text{O}}_{2}/\text{N}{\text{O}}_{2}^{-}\right)$ and nitrate $\left(\text{HN}{\text{O}}_{3}/\text{N}{\text{O}}_{3}^{-}\right)$ in liquid phase, which biochemically react with macromolecular substances in the cell (such as proteins, lipids, carbohydrates, amino acids, etc.) to change cell signaling pathways, modify genes expression, affect the response of the immune system, disrupts the cell cycle, and even induces apoptosis (24–26). Our previous study investigated the cause of leukemia cells apoptosis induced by plasma with metabonomics level, and it was inferred that plasma leads to a reduction in glutaminase activity in leukemia cells, thereby inhibiting glutamine metabolism. Glutamine metabolism provides a large amount of nutrients for tumor cells growth and proliferation, so after plasma treatment, glutamine metabolism was inhibited to eventually lead to leukemia cells apoptosis (27). In this paper, we found that when plasma jet and surface plasma were used to treat leukemia cells under the same voltage and frequency, the cell mortality of plasma jet treatment group was always higher than that of surface plasma treatment group. The qualitative and quantitative metabolites of plasma jet treatment group and surface plasma treatment group were studied by Gas Chromatography Tandem Time-of-Flight Mass Spectrometry (GC-TOFMS). At the same time, by the bioinformatics analysis of metabolites and metabolic pathways, the metabolites and metabolic pathways related with differential metabolites were screened out, and the reasons for the different effect in leukemia cells apoptosis between the two plasma generating devices were analyzed at the metabolism level.

## Method

### Surface Plasma Generation

**Figure 1:**
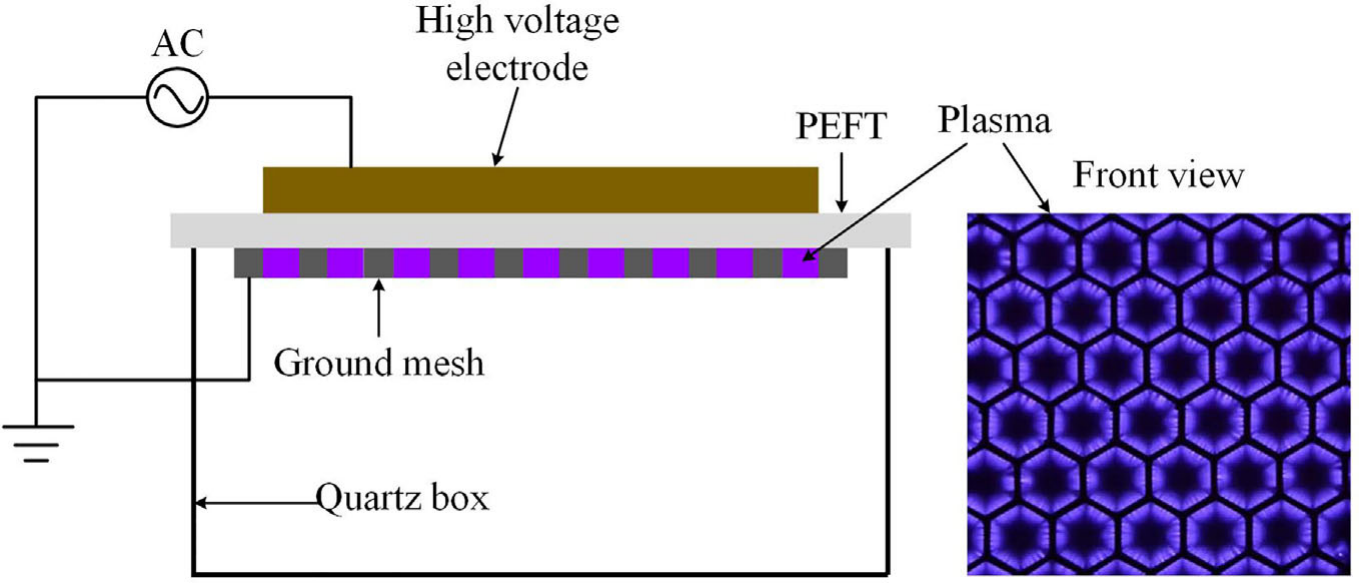
Schematic diagram and discharge photograph of surface plasma.

In this study, a surface plasma device was used to produce non-thermal plasma which has a similar configuration as reported previously (28). As shown in **Figure 1**, the plasma device consisted of a high voltage electrode, a ground electrode made of stainless-steel mesh and a 1mm Polytetrafluoroethylene (PEFT) plate sandwiched between the two electrodes. And we can see the surface plasma uniformly covers the surface of PEFT dielectric plate from **Figure 1**.

### Plasma Jet Generation

**Figure 2:**
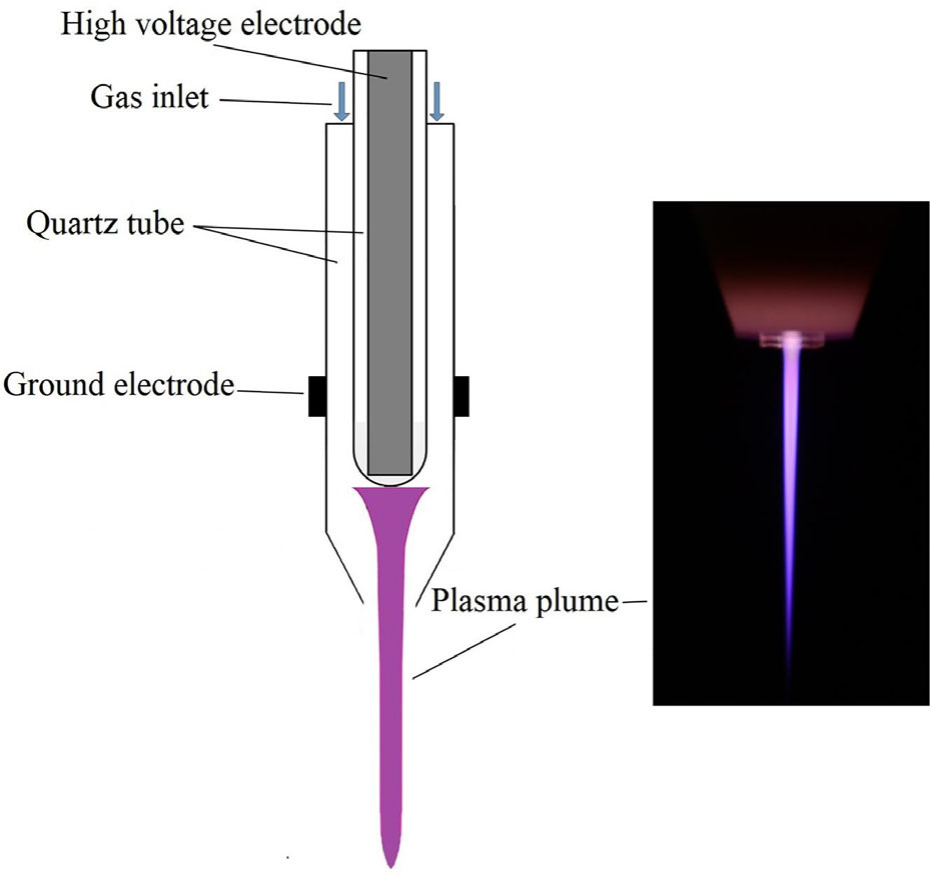
Schematic diagram and discharge photograph of plasma jet.

The structure of the plasma jet device and the photograph of plasma plume were shown in **Figure 2**. The high voltage electrode was made of a stainless-steel rod, which was sealed in a small quartz tube with a thickness of 0.75 mm. The stainless-steel rod and the quartz capillary are placed in the axis of an outer quartz tube, which has an inner and an outer diameter of 4 mm and 6 mm, respectively. The helium flowed through the device with a rate of 2 SLM, and there was a grounded electrode right below the stainless-steel rod wrapping around the outer quartz tube.

### Optical Emission Spectroscopy

We used a UV/visible spectrometer (Maya pro 2000, Ocean Optics, China) in the wavelength range of 200–800 nm to measure the emission spectrum of surface plasma and plasma jet. The optical probe was mounted directly at the discharge area of 2 cm when detecting the spectrum of surface plasma discharging. And when we detected the spectrum of plasma jet discharging, the optical probe was face-to-face with the end of plasma plume with a distance of 2 cm.

### Cell Culture Condition

The study used leukemia cell line, MOLM13. MOLM13 cells were grown in Roswell Park Memorial Institute (RPMI) 1640 medium supplemented with 10% fetal calf serum, 100 U/ml penicillin, and 50 μg/ml streptomycin (Gibco-Invitrogen, Carlsbad, CA, 15140-122).

### Cell Viability Assessment

Cell-Titer-Glo luminescent cell viability assay kit was adopted to measure cell viability, and it based on ATP participating in various enzymatic reactions in the organism maintaining normal life activities, therefore ATP is an indicator of metabolism of living cells and the production of ATP can directly reflect the number and state of cells. The stable glow signal generated by UltraGlow luciferase in the kit has a half-life of more than 5 h. Luciferase requires the participation of ATP during the light-emitting process. The light signal is proportional to the amount of ATP in the system, and ATP is directly related to the number of living cells. In this paper, 100 μl of cell sample and 100 μl of CellTiter-Glo reagent were added to an opaque 96-well plate, and the mixture was incubated at room temperature in the dark for 10 min to stabilize the fluorescence signal value, and then the opaque 96-well plate was placed into the enzyme-labeled instrument to measure its fluorescence value.

### Solvents and Reagents

RPMI 1640 medium was used to culture MOLM13 cells with 10% of fetal bovine serum (FBS). CellTIter – Glo luminescent cell viability assay kit was bought from Promega, USA. In addition, we purchased BSTFA (including 1% TMC, v/v) and methanol (HPLC grade) from Regis Technologies Inc and Anpel Laboratory Technologies Inc (Shanghai, China). Ultra-pure water we used was from ultra-pure water purifier.

### Sample Collection

We used a 24-well plate to seed 3 × 10^5^ cells/well in 300 μl of RPMI 1640 medium. Wells were treated with He plasma at 40 s as the plasma treated group, and the rest wells were treated with surface plasma at 40 s, containing 5 replicates/samples in each group. After incubation for 24 h, cells were collected and counted to ensure that the number of cells was about 1 × 10^7^ cells/sample. Cells were centrifuged at 4°C for 5 min at the speed of 1200 rpm and washed 3 times with PBS at the speed of 900 rpm. Then the cell mass in EP tube was placed in liquid nitrogen for 5 min rapidly and stored in the −80°C refrigerator until it was analyzed.

### Metabolite Extraction

Samples were transferred into the 2-ml EP tubes, and extracted with 1,000-μl extraction liquid (V_Methanol_: V_Chloroform_ = 3:1), vortex mixing for 30 s. The mixture was homogenized in ball mill for 4 min at 45 Hz, and then ultrasound treated for 5 min (incubated in ice water) repeating 3 times. After Centrifuging for 15 min at 12000 rpm, 4°C, we transferred the supernatant (800 μl) into a fresh 2-ml GC/MS glass vial, and took 40 μl from each sample and pooled them as QC sample. The extration was dried completely in a vacuum concentrator without heating, and then we added 30-μl Methoxy amination hydrochloride (20 mg/ml in pyridine) to the dried extration incubating for 30 min at 80°C. Last we added 40 μl of the BSTFA regent (1% TMCS, v/v) to the sample aliquots incubating for 1.5 h at 70°C. All samples were analyzed by gas chromatograph system coupled with a Pegasus HT time-of-flight mass spectrometer (GC-TOF-MS).

### Software of Statistical Analysis

SPSS 20.0 software was used to perform statistical analysis on cell viability and enzyme activity assessment. SIMCA software was used to perform principal component analysis (PCA) and orthogonal projections to latent structures – discriminant analysis (OPLS-DA). R language was used to visualize some results of data analysis.

## Results

### Plasma Discharging Parameter and Characters

**Figure 3:**
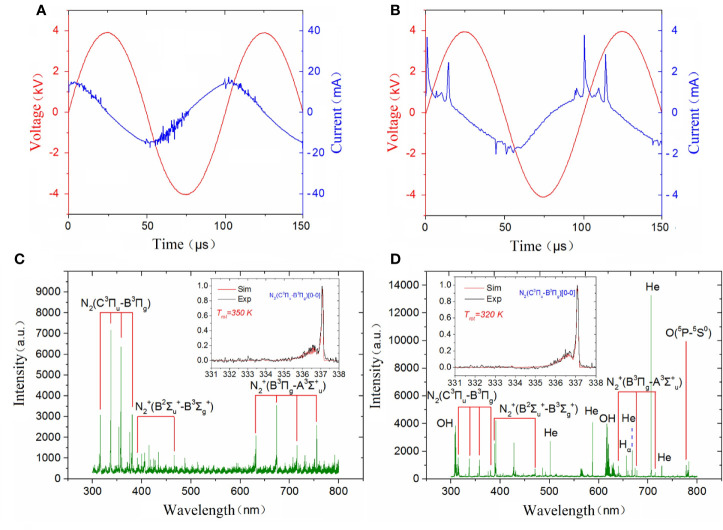
Discharge parameters of **(A)** surface plasma and **(B)** plasma jet; Emission spectra of **(C)** surface plasma and **(D)** plasma jet.

A simusoidal voltage was applied to the high-voltage electrode with a constant frequency of 10 kHz and the applied voltage was set at a peak-to-peak value of 8 kV to generate a surface plasma in ambient air, as shown in **Figure 3A**. The discharge voltage and frequency of the plasma jet was kept at 8 kV and 10 kHz and the corresponding applied voltage and current during He plasma discharge were shown in **Figure 3B**. To analyze the radiative species produced by different plasma, optical emission spectrometry (OES) diagnostics was conducted. The optical emission spectra of plasma jet and surface plasma are shown in **Figures 3C**, **D** respectively. The emission spectra in the plasma jet consists of ^•^OH transition (A_2_Σ^+^ → X_2_Π_r_) at 308 and 618 nm; He I transitions at 501.6, 587.7, 668, 706.7 and 728.2 nm; H_α_ transition at 656.5 nm; O I transitions at 777.6 nm; spectral band of the N_2_ transitions (C_3_Π_u_→B_3_Π_g_) from 316 to 350 nm, and spectral band of the ${\text{N}}_{2}^{+}$ transitions $\left({\text{B}}_{2}\Sigma_{u}^{+}\to {\text{X}}_{2}\Sigma_{g}^{+}\right)$ from 391.4 to 470 nm due to the Penning ionization. Different from plasma jet whose optical emission spectra are dominated by nitrogen and helium lines, the optical emission spectra of surface plasma are dominated by many nitrogen lines including N_2_ transitions (C_3_Π_u_→B_3_Π_g_) in the range of 300–450 nm and N transitions from 632 to 761 nm.

### Multivariate Statistical Analysis: Principal Component Analysis and Orthogonal Projections to Latent Structures—Discriminant Analysis

**Figure 4:**
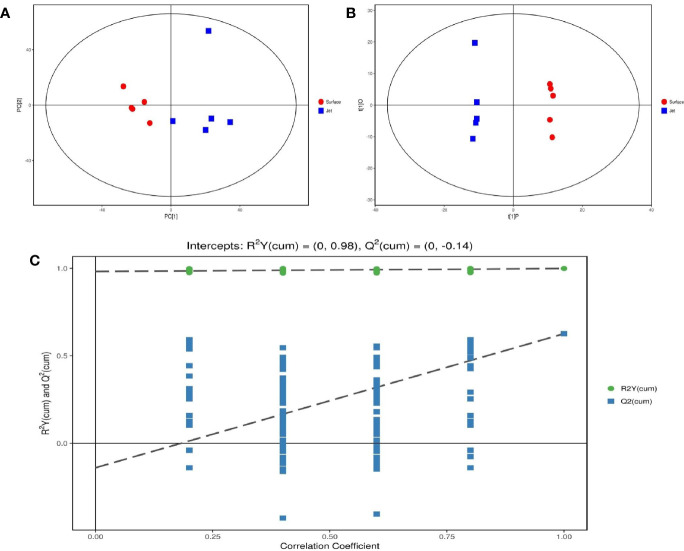
Score scatter plot of **(A)** PCA model and **(B)** OPLS-DA model; **(C)** permutation test of OPLS-DA model.

After pretreatment of qualified and quantified metabolites by Gas Chromatography Tandem Time-of-Flight Mass Spectrometry, GC-TOF-MS, we obtained data about quantity of all metabolites. We carried out a series of multivariate variable pattern recognition analysis, which were the principal component analysis (PCA) and the orthogonal least squares–discriminant analysis (orthogonal projection to latent structures-discriminant analysis, OPLS-DA). PCA result is shown in **Figure 4A**. Due to the influence of related variables, the difference variables were spread over more principal components, making it impossible to perform better visualization and subsequent analysis. Therefore, further analysis of the results was obtained by OPLS-DA, as shown in **Figure 4B**. From the result of OPLS-DA, it can be seen that two groups are significantly different, and all samples are in 95% confidence interval. The result of OPLS-DA replacement test showed that the original model had good robustness and no over-fitting phenomenon, as shown in **Figure 4C**.

### Cell Viability of Plasma Surface Plasma Versus Jet Group

**Figure 5:**
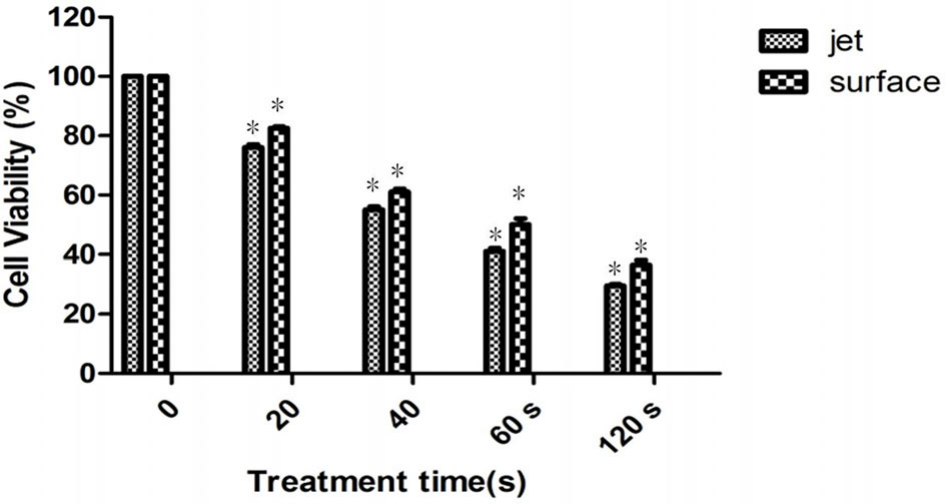
Cell viability of plasma surface group versus plasma jet group, *P < 0.05.

We totally investigated 10 samples of MOLM13 leukemia cell line, of which five samples as the experimental group were treated by He plasma jet for 40 s and the other five samples were treated by surface plasma. By cell viability assessment, our study found that when leukemia cells were treated by He plasma jet and surface plasma, respectively for the same time, cell viability in both groups gradually decreased with increasing treatment time and the cell death rate of jet treatment was much greater than that of surface treatment, as shown in **Figure 5**.

### Differential Metabolites

**Figure 6:**
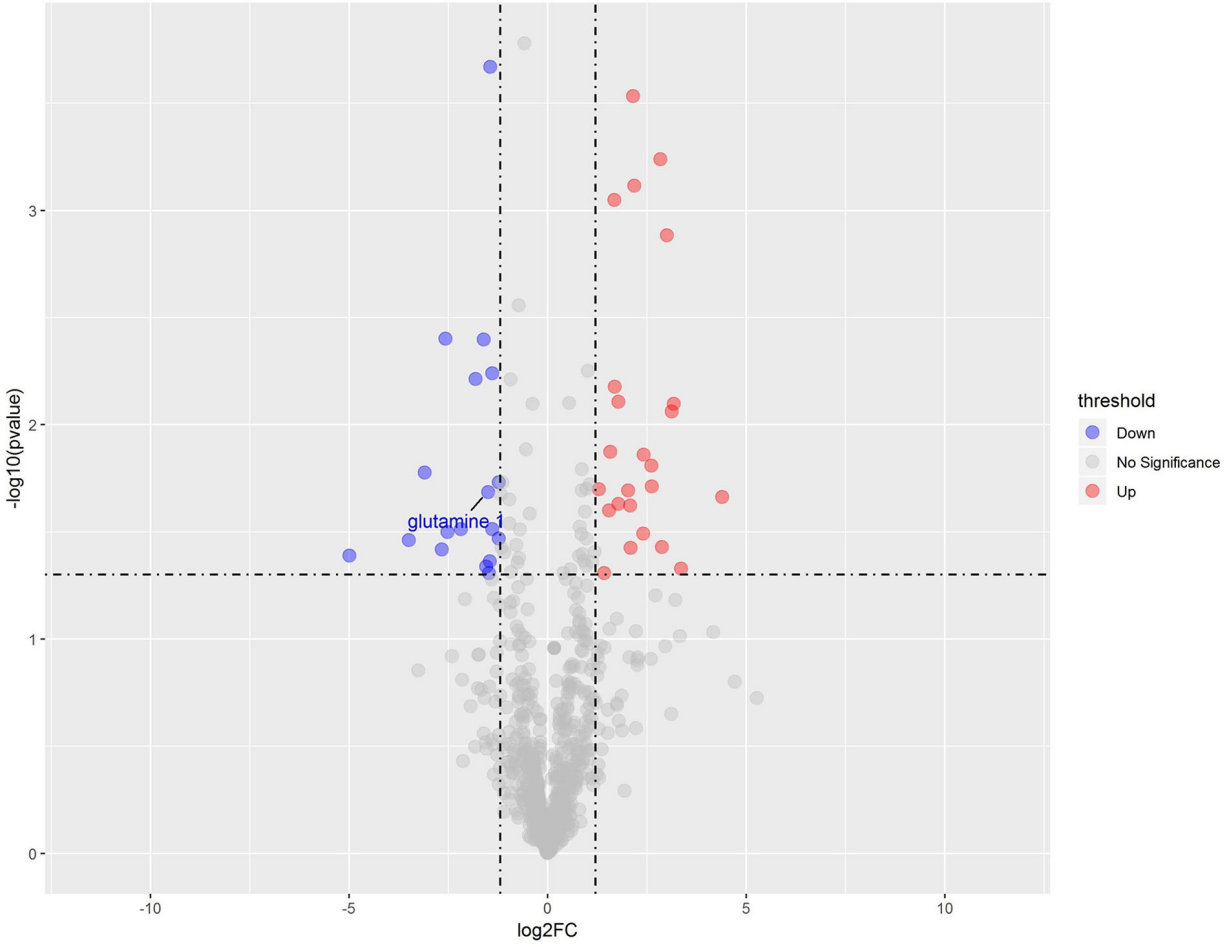
Volcano plot of differential metabolite screening.

We used a standard generally accepted by the academic community, that is, the p-value of the student’s t-test is less than 0.05, and the importance of the projection variable (VIP) of the first principal component of the OPLS-DA model is greater than 1. The differential metabolites between the jet group and the surface group were determined and further illustrated in a volcano plot (**Figure 6**). As shown in the final screening results, up-regulated metabolites were shown in red, while down-regulated metabolites were shown in blue. Glutamine was marked out as a key differential metabolite in the volcano map. It can be seen that relative quantity of glutamine in plasma surface treatment group compared with plasma jet treatment group is down-regulated.

### Cluster Analysis of Differential Metabolites

**Figure 7:**
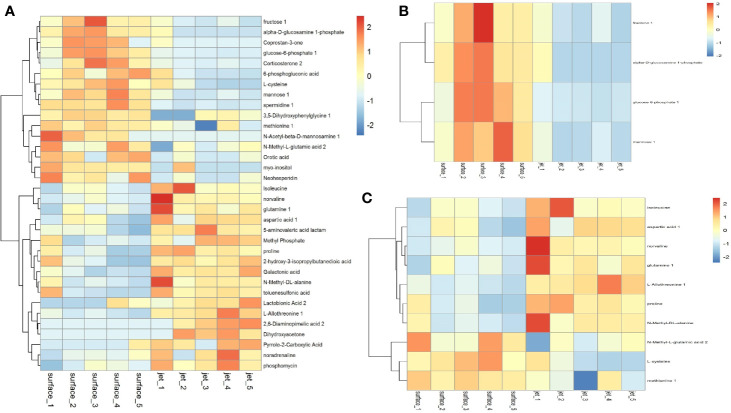
Cluster analysis of **(A)** all differential metabolites, **(B)** carbohydrate metabolites, and **(C)** amino acid metabolites.

We have screened all the up-regulated and down-regulated differential metabolites above. And the hierarchical clustering analysis will clear classify the metabolites with the same and different characteristics between the experimental groups. The results were visualized in a heatmap, as shown in **Figure 7A**. We clustered carbohydrates, amino acids respectively in different metabolites, as shown in **Figures 7B, C**. **Figure 7B** showed that carbohydrates were more up-regulated in surface plasma treatment, which might be one reason for the higher apoptosis rate of leukemia cells in plasma jet group. Glycolysis is the main metabolic pathway for the growth and rapid proliferation of tumor cells; therefore the decrease in the carbohydrate metabolites is not conducive to the growth and rapidly proliferation of tumor cells and may even cause tumor cells death. **Figure 7C** showed that the relative quantity of glutamine in plasma jet treatment group was higher than that in surface plasma treatment group, which was consistent with the result obtained when screening differential metabolites.

### Screening Metabolic Pathway Related With Differential Metabolites by KEGG

**Figure 8:**
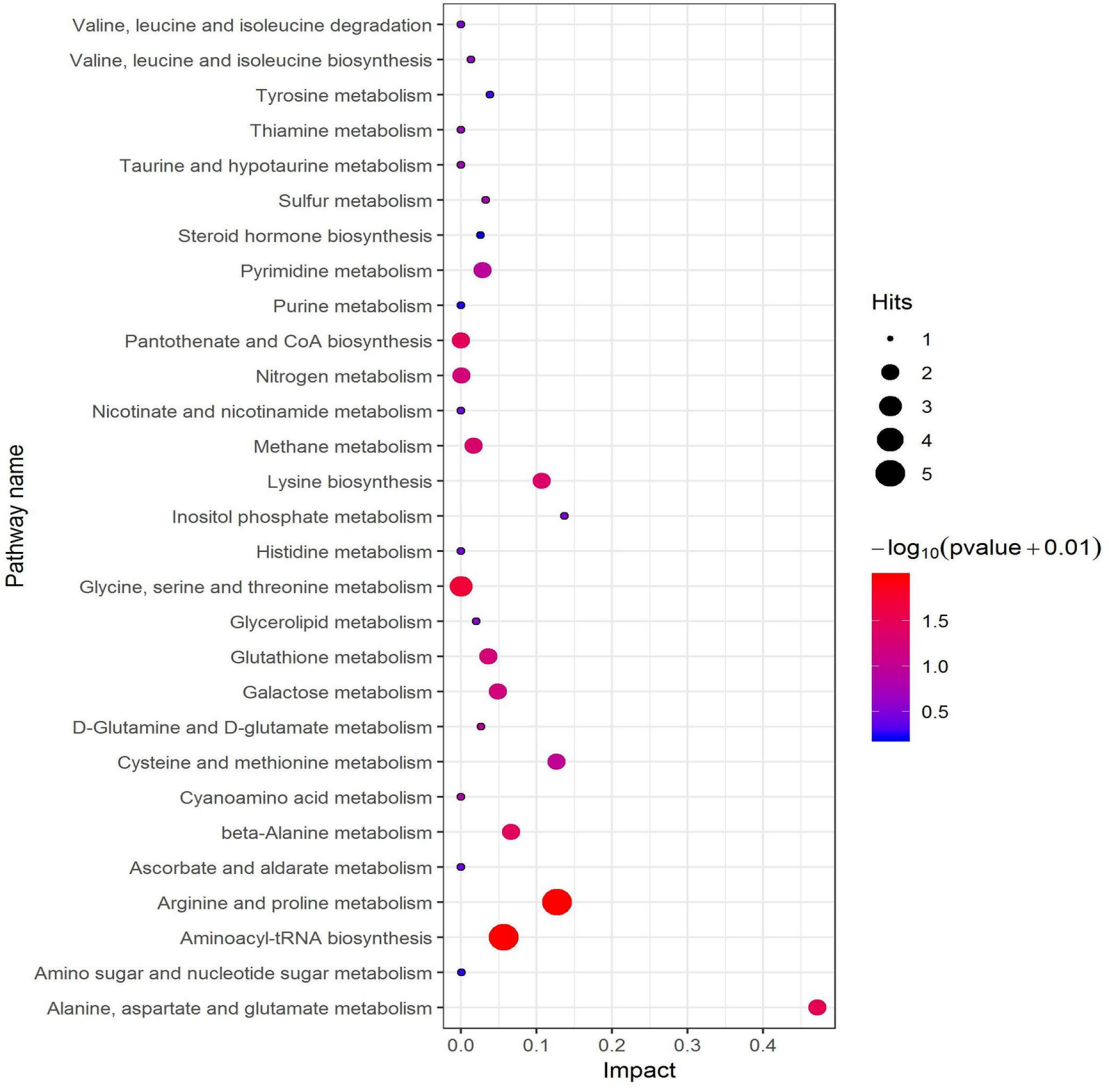
Bubble plot of metabolic pathway related with differential metabolites.

All pathways involved in differential metabolites have been found through KEGG annotation analysis. But to understand whether these pathways are closely related to experimental conditions, further metabolic pathway analysis of differential metabolites is required. Through a comprehensive analysis of the pathways of differential metabolites, including enrichment analysis and topological analysis, we further screened the pathways to find out the twenty-nine key pathways that were most relevant to metabolite differences, the first three lines of which were shown in **Table 1** and which were shown as a bubble plot in **Figure 8**. The **Table 1** showed that L-glutamine was the common differential metabolite in the top three metabolic pathways enriched in differential metabolites, and it might be the most critical differential metabolite in this paper. The results showed that the alanine, aspartate and glutamate metabolism pathway was the highest correlation with differential metabolites. Glutamine is catalyzed by glutaminase (GLS) to glutamate as a key part of the alanine, aspartate and glutamate metabolism pathway.

**Table 1:** Metabolic pathway analysis (Top 3).

Metabolic pathway	P-value	Impact	Enriched differential metabolites
Aminoacyl-tRNA biosynthesis	0.0004	0.06	L-Glutamine; L-Aspartic acid; L-Cysteine; L-Isoleucine; L-Proline
Arginine and proline metabolism	0.0005	0.13	L-Glutamine; L-Aspartic acid; L-Proline; Spermidine; Pyrrole-2-carboxylic acid
Alanine, aspartate and glutamate metabolism	0.0194	0.47	L-Glutamine; L-Aspartic acid

### Glutaminase and Glutamine Metabolism

**Figure 9:**
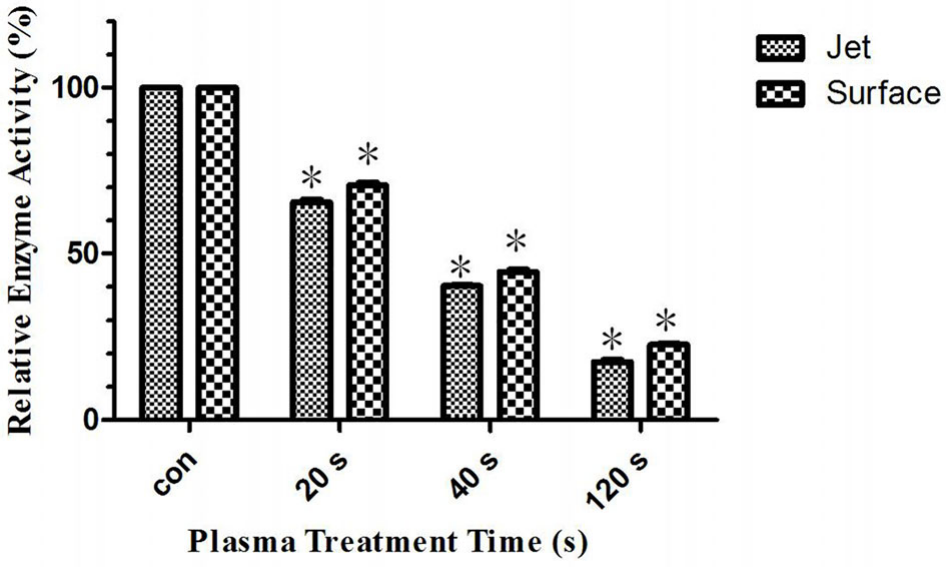
Relative glutaminase activity of He plasma jet treatment group and surface plasma treatment group, *P < 0.05.

Our previous studies have shown that glutaminase activity in leukemia cells was inhibited after plasma treatment. Therefore, glutamine metabolism was inhibited to lead to glutamine accumulation, which was a very important metabolism for tumor cells growth and rapidly proliferation. Its inhibition leads to leukemia cells apoptosis. This paper studied the reason for the difference in the effect of surface plasma and plasma jet on killing leukemia cells from metabonomics level. The result of differential metabolite analysis showed that the relative quantity of glutamine in plasma jet group was higher than that in surface plasma group, and through screening metabolic pathways with high correlation with differential metabolites, the key metabolic pathways were enriched, showing that glutamine was still the key metabolite for this experiment. Based on our previous conclusions and above research results, this paper hypothesized that the glutaminase activity of plasma jet group was lower than that of surface plasma group, so glutamine metabolism of plasma jet group was inhibited more than that of surface plasma group and then more glutamine was accumulated in plasma jet group and eventually plasma jet caused more leukemia cells apoptosis. In order to verify this hypothesis, the glutaminase activity kit was used to detect the glutaminase activity of leukemia cells after treatment with two different plasma sources, and the experimental result was shown in **Figure 9**. The glutaminase activity gradually decreases with the increase of plasma treatment time, and the glutaminase activity of plasma jet group was always lower than that of surface plasma group. Our previous studies also demonstrated that leukemia cells viability could decrease when glutaminase activity was inhibited. The above conclusion proves that the hypothesis holds, and it is proved that the plasma jet compared with surface plasma kills leukemia cells more efficiently with the same power supply voltage and frequency.

## Discussion

In recent years, plasma medicine has achieved success in many fields of application, such as bacterial killing, blood coagulation, and skin disease treatment (29–38). Research on plasma killing of cancer cells has achieved certain results. The study found that cold atmospheric plasma has a significant advantage in the treatment of tumors, that is, appropriate plasma treatment can induce tumor cell apoptosis without causing obvious damage to surrounding normal tissues, which is not available in most existing cancer treatment methods (39–41). There are many plasma devices for treatment of tumor cells currently, and the two most important devices are plasma jet and surface plasma, both of which are based on dielectric barrier discharge, but the shape and action rage of their generated plasma are different. Surface plasma source generates uniform and stable plasma on the surface of the dielectric plate, and the action range is determined by the area of the dielectric plate, while the plasma jet source generates plasma in the discharge area and plasma is ejected from the nozzle below the discharge area. The ejected plasma of plasma jet is elongated and has no fixed boundary, and the action range is small. Studies have found that these two plasma sources could effectively lead to tumor cells apoptosis and were expected to be used in clinical cancer treatment, but the effects of different plasma sources on tumor cells must be different. The application of plasma to *in vivo* treatment requires precision calculating the dose of plasma generated, so it is particularly important to study the difference on tumor killing effect under the same voltage and frequency of different plasma discharging devices. In this paper, the above two most common plasma sources were selected for research. First, the leukemia cells, MOLM13 were treated with surface plasma and plasma jet respectively, and the result showed that the mortality of leukemia cells was high increasingly with plasma treatment time increasing, and the mortality of plasma jet treatment group was always higher than that of surface plasma. In order to explore the reason why the efficiency of killing leukemia cells of plasma jet was higher than surface plasma, the qualitative and quantitative analysis of metabolites was performed on leukemia cells by the two plasma sources and the differential metabolites was screened out, and the relative quantity of differential metabolites was calculated. Then the enrichment analysis of metabolic pathways of differential metabolites was carries out, and it was found that the three metabolic pathways with high correlation with differential metabolites all have a common differential metabolite, glutamine. The above results indicated that glutamine was the most important differential metabolite in the experiment and the relative quantity of glutamine in plasma jet treatment group was higher than that in surface plasma treatment group. The glutaminase activity gradually decreased with the increase of plasma treatment time, and the glutaminase activity of plasma jet treatment group was significantly lower than that of surface plasma group. Studies have shown that tumor cells have a large metabolism dependence, which is an important difference between tumor cells and other normal cells, and one of the characteristics of this metabolic dependence is to increase the utilization rate of glutamine in anabolic pathway (42). Glutamine provides an intermediate metabolite that is lacking due to enhanced anabolism of tumor cells in TCA circle, and plays a role in maintaining the redox homeostasis of tumor cells (41–43). Therefore, glutamine metabolism is considered to be another important metabolic characteristic except for Warburg effect of tumor cells. If we clear the glutamine in tumor cells or inhibit the enzymes in the glutamine metabolism pathway, it will lead to an increase in reactive oxygen species in tumor cells, leading to tumor cells apoptosis (29, 43). The previous conclusions and the paper conclusions could explain that glutamine metabolism of plasma jet treatment group was more inhibited than that of surface plasma group, so tumor cells mortality of plasma jet group was higher than that of surface plasma group. It is also worth noting that through hierarchical clustering analysis on differential metabolites of carbohydrates and amino acids respectively, it was found that the relative quantity of metabolites in carbohydrate metabolism in plasma jet group was lower than that of surface plasma group. And this also proved that plasma jet group was not able to produce enough metabolites for energy synthesis due to its low level of glucose metabolism. Although this study was not comprehensive and has many limitations, it is the first attempt to explain the difference in the killing tumor cells effect of two different plasma sources from metabonomics level, which provides the experimental basis for the final application of these two major plasma sources in clinical cancer treatment.

## Conclusion

Plasma jet and surface plasma are two common plasma generating devices, which have excellent effects in killing tumor cells. The paper mainly analyzed the differences in the treatment effects of cancer between the two devices. The results again demonstrated that inhibition of glutamine metabolism was a metabolic abnormality produced by plasma treatment, which was a vital cause of tumor cell death. Abnormal glutamine metabolism was due to inhibition of glutaminase activity. Glutaminase activity of the He plasma jet group was lower than that of the surface plasma group, which determined that the treatment effect of the jet group was greater than that of the surface group. This study compared the ability of two major plasma generating devices to treat tumors, and analyzed the causes of the differences in the therapeutic effects of the two devices at the metabolic level, which provided a theoretical basis for others experiments to set reasonable parameters.

## Data Availability Statement

The raw data supporting the conclusions of this article will be made available by the authors, without undue reservation.

## Author Contributions

DX and MK conceived the study. YX performed the experiments and prepared the samples. NN analyzed the data and wrote the manuscript. WX participated in the experiment work. DL participated in the results discussion. HC, DX, and MK revised this manuscript. DX and NN contributed equally to this study. All authors contributed to the article and approved the submitted version.

## Funding

This work is supported by the National Natural Science Foundation of China (Grant Nos. 52077166 and 51837008), First Class of China Postdoctoral Science Foundation (2017M610639), State Key Lab oratory of Electrical Insulation and Power Equipment (EIPE18312), and Special Fund of Shaanxi Postdoctoral Science Foundation (2017BSHTDZZ04).

## Conflict of Interest

The authors declare that the research was conducted in the absence of any commercial or financial relationships that could be construed as a potential conflict of interest.

## References

[1] Virard F Sarah C Jean-Pierre C Alexis V Kémoun P Clément F. Cold Atmospheric Plasma Induces a Predominantly Necrotic Cell Death via the Microenvironment. PLoS One (2015) 10(8):e0133120. 10.1371/journal.pone.0133120 26275141 PMC4537210

[2] Gay-Mimbrera J García MC Isla-Tejera B Rodero-Serrano A García-Nieto AV Ruano J. Clinical and Biological Principles of Cold Atmospheric Plasma Application in Skin Cancer. Adv Ther (2016) 33(6):894–909. 10.1007/s12325-016-0338-1 27142848 PMC4920838

[3] Xu D Cui Q Xu Y Wang B Tian M. Systemic study on the safety of immuno-deficient nude mice treated by atmospheric plasma-activated water. Plasma Sci Technol 20(4):044003. 10.1088/2058-6272/aa9842

[4] Dai X Bazaka K Thompson EW Ostrikov KK. Cold Atmospheric Plasma: A Promising Controller of Cancer Cell States. Cancers (2020) 12(11):3360. 10.3390/cancers12113360 PMC7696697 33202842

[5] Yan D Talbot A Nourmohammadi N Cheng X Canady J Sherman J. Principles of using Cold Atmospheric Plasma Stimulated Media for Cancer Treatment. Sci Rep (2015) 5(5):18339. 10.1038/srep18339 26677750 PMC4683589

[6] Yan D Sherman JH Keidar M. Cold atmospheric plasma, a novel promising anti-cancer treatment modality. Oncotarget (2017) 8(9):15977–95. 10.18632/oncotarget.13304 PMC5362540 27845910

[7] Gjika E Pal-Ghosh S Tang A Kirschner M Tadvalkar G Canady J. Adaptation of Operational Parameters of Cold Atmospheric Plasma for in Vitro Treatment of Cancer Cells. ACS Appl Materials Interfaces (2018) 10(11):9269. 10.1021/acsami.7b18653 PMC5954411 29473408

[8] Daeschlein G Hillmann A Gumbel D Sicher C Von-Podewils S Stope MB. Enhanced anticancer efficacy by drug chemotherapy and cold atmospheric plasma against melanoma and glioblastoma cell lines in vitro. IEEE Trans Radiat Plasma Med Sci (2018) 2(2):153–9. 10.1109/TRPMS.2018.2789659

[9] Xu D Xu Y Cui Q Liu D Liu Z Wang X. Cold atmospheric plasma as a potential tool for multiple myeloma treatment. Oncotarget (2018) 9(26):18002–17. 10.18632/oncotarget.24649 PMC5915053 29719586

[10] Denis G Sander B Nadine G Matthias N Axel E Axel K. Cold Atmospheric Plasma in the Treatment of Osteosarcoma. Int J Mol Sci (2017) 18(9):2004. 10.3390/ijms18092004 PMC5618653 28925941

[11] Keidar M Shashurin A Volotskova O Stepp MA Srinivasan P Sandler A. Cold Atmospheric Plasma in Cancer Therapy. Phys Plasmas (2013) 20(5):S169–80. 10.1063/1.4801516

[12] Isbary G Shimizu T Li Y Stolz W Thomas HM Morfill GE. Cold atmospheric plasma devices for medical issues. Expert Rev Med Devices (2013) 10(3):367–77. 10.1586/erd.13.4 23668708

[13] Thiyagarajan M Anderson H Gonzales X. Induction of apoptosis in human myeloid leukemia cells by remote exposure of resistive barrier cold plasma Biotechnol Bioeng (2014) 111(3):565–74. 10.1002/bit.25114. 24022746

[14] Stephanie A Arndt S Wacker E Li Y Shimizu T Thomas HM Morfill GE. Cold atmospheric plasma, a new strategy to induce senescence in melanoma cells. Exp Dermatol (2013) 22(4):284–9. 10.1111/exd.12127 23528215

[15] Akhlaghi M Rajaei H Mashayekh AS Shafiae M Mahdikia H Khani M. Determination of the optimum conditions for lung cancer cells treatment using cold atmospheric plasma. Phys Plasmas (2016) 23(10):1409–15. 10.1063/1.4964899

[16] Barekzi N Laroussi M. Dose-dependent killing of leukemia cells by low-temperature plasma. J Phys D Appl Phys (2012) 45(42):422002. 10.1088/0022-3727/45/42/422002

[17] Thiyagarajan M Waldbeser L Whitmill A. THP-1 leukemia cancer treatment using a portable plasma device. Stud Health Technol Inform (2012) 173:515–7 10.3233/978-1-61499-022-2-515. 22357047

[18] Schneider C Lisa G Stephanie A Sigrid K Zimmermann JL Fischer MJM. Cold atmospheric plasma causes a calcium influx in melanoma cells triggering CAP-induced senescence. Sci Rep (2018) 8(1):10048. 10.1038/s41598-018-28443-5 29968804 PMC6030087

[19] Xia J Zeng W Xia Y Wang B Xu D Liu D. Cold Atmospheric Plasma Induces Apoptosis of Melanoma Cells via Sestrin2-mediated iNOS Signaling. J Biophotonics (2018) 12(1) e201800046–. 10.1002/jbio.201800046 29931745

[20] Sensenig R Kalghatgi S Cerchar E Fridman G Shereshevsky A Torabi B. Retraction note to: Non-thermal plasma induces apoptosis in melanoma cells via production of intracellular reactive oxygen species. Ann Biomed Eng (2011) 39(2):674–87. 10.1007/s10439-010-0197-x PMC3268344 21046465

[21] Laroussi M. Low-Temperature Plasma Jet for Biomedical Applications: A Review. IEEE Trans Plasma Sci (2015) 43(3):703–12. 10.1109/TPS.2015.2403307

[22] Hiromasa T Masaaki M Kenji I Shinya T Hiroaki K Fumitaka K. New Hopes for Plasma-Based Cancer Treatment. Plasma (2018) 1(1):150–5. 10.3390/plasma1010014

[23] Metelmann HR Seebauer C Rutkowski R Schuster M Bekeschus S Metelmann P. Treating cancer with cold physical plasma: On the way to evidence-based medicine. Contributions to Plasma Phys (2018) 58:415–9. 10.1002/ctpp.201700085

[24] Shen J Zhang H Xu Z Zhang Z Chu PK. Preferential production of reactive species and bactericidal efficacy of gas-liquid plasma discharge. Chem Eng J (2019) 362:402–12. 10.1016/j.cej.2019.01.018

[25] Zhang Z Shen J Cheng C Zimu X Xia W. Generation of reactive species in atmospheric pressure dielectric barrier discharge with liquid water. Plasma Sci Technol (2018) 20(4):044009. 10.1088/2058-6272/aaa437

[26] Liu K Lei J Zheng Z Zhu Z Liu S. The hydrophilicity improvement of polytetrafluoroethylene by Ar plasma jet: The relationship of hydrophilicity, ambient humidity and plasma parameters. Appl Surface Sci (2018) 458(NOV.15):183–90 10.1016/j.apsusc.2018.07.061.

[27] Xu D Taylor D Zimmermann JL Bunk W Monetti R Isbary G. Effect of cold atmospheric plasma treatment on the metabolites of human leukemia cells. Cancer Cell Int (2019) 19(1):135. 10.1186/s12935-019-0856-4 31130824 PMC6525389

[28] Ma RN Feng HQ Liang YD Zhang Q Tian Y Su B. An atmospheric-pressure cold plasma leads to apoptosis in Saccharomyces cerevisiae by accumulating intracellular reactive oxygen species and calcium. J Phys D Appl Phys (2013) 46(28):285401. 10.1109/PLASMA.2013.6634816.

[29] Li YF Taylor D Zimmermann JL Bunk W Monetti R Isbary G. In vivo skin treatment using two portable plasma devices: Comparison of a direct and an indirect cold atmospheric plasma treatment. Clin Plasma Med (2013) 1(2):35–9. 10.1016/j.cpme.2013.09.001

[30] Bekeschus S Kolata J Winterbourn C Kramer A Turner R Weltmann KD. Hydrogen peroxide: A central player in physical plasma-induced oxidative stress in human blood cells. Free Radical Res (2014) 48(5):542. 10.3109/10715762.2014.892937 24528134

[31] Zhu W Keidar M Zhang LG. Enhanced human bone marrow mesenchymal stem cell chondrogenic differentiation on cold atmospheric plasma modified cartilage scaffold. Mrs Proc (2014) 1723:28–33 10.1557/opl.2014.940.

[32] Babington P Rajjoub K Canady J Siu A Keidar M Sherman JH. Use of cold atmospheric plasma in the treatment of cancer. Biointerphases (2015) 10(2):029403. 10.1116/1.4915264 25791295

[33] Vermeylen S Waele JD Vanuytsel S Backer JD Jonas VDP Ramakers M. Cold atmospheric plasma treatment of melanoma and glioblastoma cancer cells. Plasma Processes Polymers (2016) 13(12):1195–205. 10.1002/ppap.201600116

[34] Cheng X Rajjoub K Shashurin A Yan D Sherman J Bian K. Enhancing cold atmospheric plasma treatment of cancer cells by static magnetic field. Bioelectromagnetics (2017) 38(1):53–62. 10.1002/bem.22014 27748977

[35] Mateu-Sanz M Tornin J Ginebra M Canal C. Cold Atmospheric Plasma: A New Strategy Based Primarily on Oxidative Stress for Osteosarcoma Therapy. J Clin Med (2021) 10(4):893. 10.3390/jcm10040893 33672274 PMC7926371

[36] Kim JY Ballato J Foy P Hawkins T Wei Y Li J. Apoptosis of lung carcinoma cells induced by a flexible optical fiber-based cold microplasma. Biosensors Bioelectronics (2011) 28(1):333–8. 10.1016/j.bios.2011.07.039 21820891

[37] Isbary G. “Cold Atmospheric Plasma for Clinical Purposes: Promising Results in Patients and Future Applications”. In: Machala Z Hensel K Akishev Y, editors. Plasma for Bio-Decontamination, Medicine and Food Security. NATO Science for Peace and Security Series A: Chemistry and Biology. Dordrecht: Springer (2012). 10.1007/978-94-007-2852-3_24

[38] Ratovitski EA Cheng X Yan D Sherman JH Canady J Trink B. Anti-Cancer Therapies of 21st Century: Novel Approach to Treat Human Cancers Using Cold Atmospheric Plasma. Plasma Processes Polymers (2015) 11(12):1128–37. 10.1002/ppap.201400071

[39] Graves DB Graves DB. Reactive Species from Cold Atmospheric Plasma: Implications for Cancer Therapy. Plasma Processes Polymers (2015) 11(12):1120–7. 10.1002/ppap.201400068

[40] Keidar M. “Cold plasma application in cancer therapy”. In: 2016 IEEE International Conference on Plasma Science (ICOPS). IEEE (2016). 10.1109/PLASMA.2016.7534292

[41] Li T Le A. Glutamine Metabolism in Cancer. In: Le A, editor. The Heterogeneity of Cancer Metabolism. Advances in Experimental Medicine and Biology, vol 1063. Cham: Springer. (2018). 10.1007/978-3-319-77736-8_2 29946773

[42] Goto M Miwa H Shikami M Tsunekawa-Imai N Suganuma K Mizuno S. Importance of Glutamine Metabolism in Leukemia Cells by Energy Production Through TCA Cycle and by Redox Homeostasis. Cancer Invest (2014) 32(6):241–7. 10.3109/07357907.2014.907419 24762082

[43] Polet F Corbet C Pinto A Rubio LI Martherus R Bol V. Reducing the serine availability complements the inhibition of the glutamine metabolism to block leukemia cell growth. Oncotarget (2016) 7(2):1765–76. 10.18632/oncotarget.6426 PMC4811496 26625201

